# The NICOLA questionnaire trial (NICOLA-QT): a randomised trial of the effect of timing and mode of delivery on the completion and return of a self-assessment questionnaire

**DOI:** 10.1186/1745-6215-16-S2-P107

**Published:** 2015-11-16

**Authors:** Lisa Maguire, Frances Burns, Mike Clarke

**Affiliations:** 1 Queen's University Belfast, Belfast, UK; 2 University of Liverpool, Liverpool, UK

## Background

NICOLA-QT is designed to detect the optimal time and delivery method for giving a self-assessment questionnaire to participants, seeking to overcome the low response rates associated with these. The outline has been registered as SWAT-2. NICOLA-QT is embedded in the NICOLA cohort study, which is recruiting 8,500 people over 50 years of age in Northern Ireland and follow them into old age, making a comprehensive assessment of their health, lifestyles and their decision making.

## Aim

The overall aim of NICOLA-QT is to examine the impact of differing times and formats of a self-assessment questionnaire on completion rates.

## Methods

Participants were randomly allocated to receive a questionnaire by either: being handed it in person at the end of a home visit or in the post approximately one week after their home visit, along with a thank-you certificate for participation to-date.

## Results

The primary outcome measure is whether or not the self-completion questionnaire is returned. Secondary outcome measures include the time taken to return the questionnaire and the completeness of the questionnaire. Recruitment for the NICOLA cohort began in January 2014 and is expected to continue until late 2015. Although the cohort is still recruiting, this presentation will detail preliminary findings from the interim analysis of 4000 participants in NICOLA-QT.

## Conclusion

This SWAT (Study Within A Trial) aims to provide evidence on what research participants might prefer when asked to complete a questionnaire, specifically examining low-cost adjustments that researchers can use to improve return rates.

## Trial Registration

NCT01978522

